# Effect of dextran-70 on outcome in severe sepsis; a propensity-score matching study

**DOI:** 10.1186/s13049-017-0413-x

**Published:** 2017-07-06

**Authors:** Peter Bentzer, Marcus Broman, Thomas Kander

**Affiliations:** 1 Department of Anesthesia and Intensive Care Helsingborg Hospital, Helsingborgs lasarett, Charlotte Yhlens gata 10, 251 87 Helsingborg, Sweden; 2 Department of Clinical Sciences Lund, Lund University, Box 157, 221 00 Lund, Sweden; 3 Department of Intensive and Perioperative Care, Skåne University Hospital Lund, Getingevägen, 221 85 Lund, Sweden

**Keywords:** Acute kidney injury, Dextran, Colloid, Crystalloid, Sepsis, Resuscitation

## Abstract

**Background:**

Albumin may be beneficial in patients with septic shock but availability is limited and cost is high. The objective of the present study was to investigate if the use of dextran-70 in addition to albumin and crystalloids influences organ failure or mortality in patients with severe sepsis or septic shock.

**Methods:**

Patients with severe sepsis or septic shock (*n* = 778) admitted to a university hospital intensive care unit (ICU) between 2007 and 2015 that received dextran-70 during resuscitation were propensity score matched to controls at a 1 to 1 ratio. Outcomes were highest acute kidney injury network (AKIN) score the first 10 days in the ICU, use of renal replacement therapy, days alive and free of organ support the first 28 days after admission to ICU, mortality and events of severe bleeding. Outcomes were assessed using paired hypothesis testing.

**Results:**

Propensity score matching resulted in two groups of patients with 245 patients in each group. The dextran group received a median volume of 1483 ml (interquartile range, 1000–2000 ml) of dextran-70 during the ICU stay. Highest AKIN score did not differ between the control- and dextran groups (1 (0–3) versus 2 (0–3), *p* = 0.06). Incidence of renal replacement therapy in the control- and dextran groups was similar (19% versus 22%, *p* = 0.42, absolute risk reduction −2.9% [95% CI: −9.9 to 4.2]). Days alive and free of renal replacement, vasopressors and mechanical ventilation did not differ between the control- and dextran groups. The 180-day mortality was 50.2% in the control group and 41.6% in the dextran group (*p* = 0.046, absolute risk reduction 8.6% [−0.2 to 17.4]). Fraction of patients experiencing a severe bleeding in the first 10 days in the ICU did not differ between the control and dextran groups (14% versus 18%, *p* = 0.21).

**Discussion:**

There is a paucity of high quality data regarding effects of dextran solutions on outcome in sepsis. In the present study, propensity score matching was used in attempt to reduce bias.

**Conclusion:**

No evidence to support a detrimental effect of dextran-70 on mortality or on organ failures in patients with severe sepsis or septic shock could be detected.

**Electronic supplementary material:**

The online version of this article (doi:10.1186/s13049-017-0413-x) contains supplementary material, which is available to authorized users.

## Background

The optimal fluid therapy for patients with severe sepsis and septic shock is debated [1–3]. Colloids have theoretical advantages compared to crystalloids because they are more efficacious plasma expanders than crystalloids and may minimize harmful effects of fluid overload [4–11]. Some support for the use of albumin as an adjunct to the crystalloids may be inferred from the subgroup analyses of data from two large randomized trials showing improved outcomes in patients that received albumin compared to those treated only with saline [12, 13]. Moreover, the surviving sepsis guidelines support the use of albumin in patients requiring large amounts of fluid for hemodynamic stabilisation [14]. However, albumin is expensive, availability is limited and transfer of viruses remains a possibility in albumin products derived from human donors. Taken together, this provides a rational for the study of alternatives to albumin in patients with sepsis requiring large amounts of fluid.

Given that the use of hydroxyletyl starches (HES) in septic patients is discouraged [15–18] dextrans are a group of colloids that are of potential interest. Dextrans are branched glucose polysaccharides and dextran-70 is a more efficacious plasma volume expander than albumin [8, 19, 20]. In addition, dextrans possess antithrombotic and rheological effects [21]. Two small studies have suggested that the use of dextran-70 in sepsis may be associated with increased bleeding and increased risk of acute kidney injury thus raising safety concerns [22, 23]. In attempt to further investigate safety of dextran-70 as an alternative to albumin we propensity-score matched patients with severe sepsis or septic shock who received dextran-70 to those who did not receive dextran-70 in a cohort of patients treated in a single intensive care unit (ICU). Effects of dextran-70 on measures of organ failure, on incidence of severe bleeding and on mortality were then investigated.

## Methods

### Subjects

The study was approved by the regional ethical vetting board in Lund (registration number 2014/916). Patients admitted to the general tertiary ICU at Lund University Hospital, Sweden between 1 of January 2007 and 9 of November 2015 with the diagnosis of severe sepsis (ICD-code R65.1) or septic shock (ICD-code R57.2) according to Sepsis-2 definition [24] were eligeble for inclusion. Patients <18 years of age and patients who received hydroxyethyl starch or gelatin during resuscitation were excluded. To increase power to detect effects on renal function, patients receiving renal replacement therapy (RRT) prior to admission were excluded. The manuscript was prepared according to the STROBE guidelines for observational studies [25].

Patients with severe sepsis and septic shock were identified using data from the Swedish Intensive Care registry (SIR). For patients with more than 1 admission with the diagnosis of severe sepsis or septic shock only the first admission was included in the analysis. Mortality data was imported from SIR. Physiological and laboratory data and pre-existing conditions (age, gender, chronic obstructive pulmonary disease (COPD), renal failure, diabetes), outcome variables (except mortality) and fluid administration data were collected from raw data, i.e. from the electronic master chart system of the hospital or from the patient data management system at the ICU. Patients were divided into a dextran and a control group, based on whether they received dextran-70 or not the first 5 days of the ICU-stay. The control group was resuscitated with a combination of crystalloids and 5 and 20% albumin. The use of dextran-70 (6% dextran solution with a mean molecular weight of 70 kilodalton [kDa] dissolved in 0.9% sodium chloride, Macrodex®, Meda) during the resuscitation was not regulated in local guidelines and was left to the discretion of the attending physician. Dextran-1 (Promiten®, Meda) was given prior to dextran-70 as a prophylaxis against anaphylaxis.

A secondary sensitivity analysis in which effects of a higher dose of dextran-70 was investigated was planned a priori. In this analysis only patients receiving >900 ml dextran-70 the first 5 days after admission (*n* = 323) were available for propensity score matching in the dextran group. These patients were propensity score matched to the same non-dextran group (control group) as in the primary analysis. Patients treated with ≤900 ml dextran-70 were excluded in this secondary analysis. The rationale for choosing >900 ml as a cut off for this analysis was that we wanted include patients that received ≈ two 500 ml bags of dextran-70 or more and the priming of the pumps usually resulted in slightly less than 1000 ml of dextran being given. According to the Summary of Product Characteristics for Macrodex®, maximum daily dose is 2500 ml.

### Outcomes

Maximum acute kidney injury score according to the Acute Kidney Injury Network (AKIN) criteria [26] during the first 10 days of admission to the ICU was used as an outcome reflecting renal effects of dextran-70. The rationale for choosing this time frame was that dextran-70 is mainly administered during the first few days of admission to the ICU and that renal failure after day 10 is likely to be increasingly influenced by factors other than dextran administration. Other outcomes were use of RRT, days alive and free of RRT, days alive and free of mechanical ventilation, days alive and free of vasopressor therapy during the first 28 days of the ICU stay, and 28, 90 and 180-day mortality. Any patient that died during the 28-day observation period was assigned 0 days alive and free of any organ support. To assess potential effects of dextran on incidence of severe bleeding episodes patients that received more than 3 units of packed red blood cells at any day during the first 10 days in the ICU were defined as having experienced a severe bleeding episode.

### Statistical analyses

Dextran- and non-dextran-treated patients were propensity score matched to adjust for differences in baseline variables associated with outcome. The propensity score was calculated with linear logistic regression using a one_to_many macro for SAS [27] with the covariates specified in Table 1. Physiological and laboratory variables used in the propensity score matching were collected within 90 min of admission to the intensive care unit. A greedy matching procedure matched treated to controls at a ratio of 1:1. In a first step a match was sought with a propensity score that was identical to 8 decimal places to the treated patient. If no match was found, a match would be sought at 7 decimal places and so on. If no match was found at 1 decimal place, the patient receiving dextran-70 was excluded from the study. A control could only be used once. The standardized difference was used as a balance diagnostics as it is not confounded by sample size [28]. A standardized difference of ≤10% is suggested to indicate negligible differences in the mean or prevalence of covariates between groups [29].

**Table 1:** Patient demographics before and after propensity matching

	Unmatched groups		Standardized difference	*P*-value	Propensity-matched groups		Standardized difference	*P*-value
	Control *N* = 342	Dextran *N* = 436			Control *N* = 245	Dextran *N* = 245		
Pre-existing conditions								
Age, mean (SD^a^)	61.4 (17)	66.0 (15)	0.29	0.0001	63.6 (16)	63.7 (16)	0.009	0.92
Male gender, no (%)	140 (41)	198 (45)	0.09	0.21	102 (42)	103 (42)	0.08	0.93
Blood malignancy^b^, no (%)	58 (17.0)	20 (4.6)	0.41	0.0001	18 (7)	17 (17)	0.02	0.86
COPD^c^, no (%)	39 (11)	51 (12)	0.0092	0.89	32 (13)	32 (13)	0.00	1.00
Chronic renal failure, no (%)	14 (4.1)	10 (2.3)	0.10	0.15	10 (4.1)	9 (3.7)	0.02	0.82
Cirrhosis, no (%)	15 (4.4)	11 (2.5)	0.10	0.15	10 (4.1)	10 (4.1)	0.00	1.00
Diabetes, no (%)	38 (11)	45 (10)	0.03	0.72	28 (11)	31 (13)	0.04	0.68
Immunosuppression^d^, no (%)	54 (16)	38 (8.7)	0.22	0.002	26 (10.6)	22 (9.0)	0.05	0.54
Malignancy^e^, no (%)	46 (13)	62 (14)	0.02	0.76	32 (13)	32 (13)	0.00	1.00
Nosocomial infection^f^, no (%)	44 (13)	31 (7)	0.19	0.007	21 (8.6)	23 (9.4)	0.03	0.75
Surgery^g^, no (%)	71 (21)	92 (21)	0.008	0.91	51 (21)	51 (21)	0.00	1.00
GI^h^ bleeding, no (%)	3 (0.9)	3 (0.7)	0.02	0.77	1 (0.4)	2 (0.8)	0.05	0.56
DIC^i^, no (%)	39 (11)	35 (8)	0.11	0.11	20 (8)	20 (8)	0.00	1.00
I.C.^j^ volume effect, no (%)	5 (1.5)	0 (0)	0.17	0.01	0 (0)	0 (0)	0.00	1.00
Airway infection, no (%)	94 (28)	106 (24)	0.07	0.32	66 (27)	59 (24)	0.07	0.93
Physiological and laboratory variables at admission^k^, mean (SD)								
Heart rate, mean (SD)	110 (24)	110 (24)	0.04	0.60	110 (24)	112 (25)	0.08	0.35
SBP^l^, (mmHg)	111 (30)	103 (28)	0.04	0.60	108 (29)	108 (29)	0.006	0.95
Lactate (mmol/L)	4.0 (3.8)	2.9 (3.1)	0.06	0.42	3.8 (3.4)	3.8 (3.1)	0.03	0.76
Norepinephrine (μg/min)	3.7 (5.7)	5.4 (8.9)	0.24	0.002	4.0 (5.7)	3.8 (5.5)	0.03	0.76
Temperature (°Celcius)	37.3 (1.6)	37.4 (1.2)	0.07	0.30	37.3 (1.4)	37.3 (1.2)	0.008	0.93
Oxygenation points^m^	2.0 (1.1)	1.9 (1.1)	0.02	0.79	2.0 (1.1)	2.0 (1.1)	0.02	0.84
Leucocytes (× 10^9^/L)	16.6 (39.3)	14.0 (18.7)	0.09	0.22	15.5 (23.6)	15.2 (23.6)	0.01	0.89
Platelets (× 10^9^/L)	151 (133)	184 (119)	0.26	0.0003	173 (138)	167 (106)	0.05	0.61
pH	7.34 (0.14)	7.32 (0.12)	0.12	0.09	7.34 (0.13)	7.34 0.11)	0.01	0.91
Bilirubin (μmol/L)	31.0 (49.7)	20.8 (29.9)	0.25	0.0006	23.6 (26.3)	24.6 (37.8)	0.03	0.73
Creatinine (μmol/L)	173 (136)	174 (133)	0.006	0.94	180 (137)	180 (140)	0.003	0.97

^a^Standard deviation

^b^Lymphoma, acute leukaemia or myeloma

^c^Chronic obstructive pulmonary disease

^d^Chronic steroid treatment correlative to ≥0.3 mg/kg prednisolone/day, radiation, or chemo therapy

^e^Cancer spread beyond the regional lymph nodes

^f^Infection that developed after ≥48 h in hospital or secondary to surgical or medical procedure

^g^Before admission to intensive care

^h^Gastro-intestinal

^i^Disseminated intravascular coagulopathy

^j^Intra-cranial

^k^First value within 90 min after admission except for “Norepinephrine” which is the mean dose until the first day’s morning

^l^Systolic blood pressure

^m^In accordance with SAPS 3. 1 point: PaO_2_ ≥ 8 kPa and spontaneous breathing (SB). 2 points PaO_2_ ≤ 8 kPa and SB. 3 points PaO_2_/FiO_2_ ≥ 13.3 and mechanical ventilation (MV). 4 points PaO_2_/FiO_2_ ≤ 13.3 and MV

Sample size was based on the number of available patients during the study period. Variables were summarized using mean or median with standard deviation or range as distribution measurement. An independent statistician performed propensity score matching using SAS version 9.4 (SAS Institute Inc., Cary, NC, USA) prior to any comparison between the groups. Kaplan-Meier survival analysis was performed and is presented in graphs with corresponding stratified log-rank test. In accordance with previous recommendations [30, 31] all comparisons between the groups after propensity score matching was performed using paired hypothesis testing. Wilcoxon rang sum test was used for continuous variables and McNemar’s test for categorical variables by SPSS Statistics version 24 (SPSS Inc., Chicago, Ill., USA). A two-sided *P* value of less than 0.05 was considered to indicate statistical significance.

## Results

**Fig. 1:**
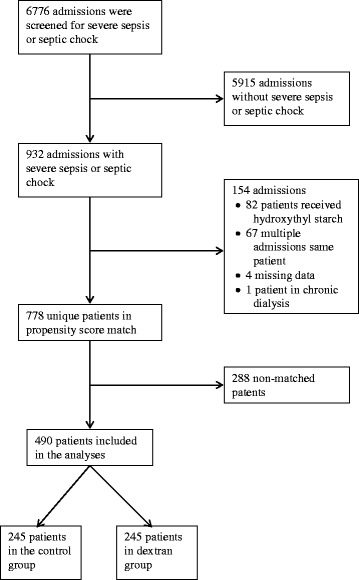
Consort scheme of the study patients

A Consort chart of patients is presented in Fig. 1. Of 6776 admissions, 932 (13.8%) were diagnosed with severe sepsis or septic shock and a total of 342 control patients and 436 dextran-70 treated patients were eligible for inclusion in the propensity score match. At total of 490 of these patients were matched at a 1:1 ratio, i.e. 245 unique patients in the control group and 245 unique patients in the dextran group. The median number of propensity score matched patients included each year was 24 (min-max range 14–48) in the control group and 28 (min-max range 20–33) in the dextran group. For number of patients included in the dextran and control groups stratified by year of admission see Additional file 1.

Baseline demographics, pre-existing medical conditions, and clinical, physiologic, and laboratory data in the unmatched and matched study population are summarized in Table 1. Matching reduced standardized differences between the groups in baseline variables to ≤10% for all variables. The dose of dextran-70 in the dextran group the first day was 1000 ml (interquartile range 500–1000 ml) and for the first 5 days 399 ml/day (interquartile range 200–656 ml/day) (Table 3). Cumulative dose of dextran-70 for the ICU stay was 1483 ml (interquartile range 1000–2000 ml), which corresponds to 17 ml/kg (interquartile range 12–27 ml/kg).

**Fig. 2:**
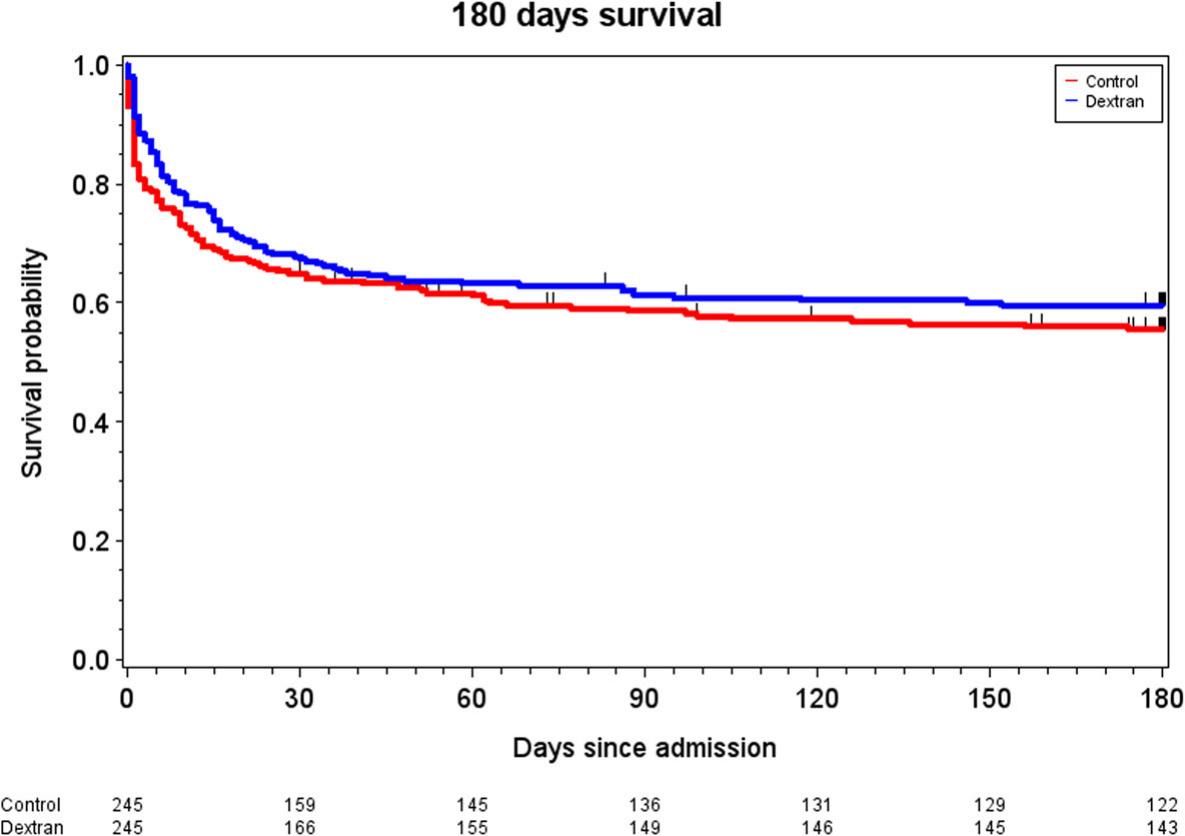
Kaplan–Meier estimates of the probability of 180-day survival. *P* = 0.28 for the comparison between the control group (*red line*) and the dextran group (*blue line*). Difference between groups was tested using the stratified log-rank test

There was a signal for a higher maximal AKIN score in the dextran group than in the control group (*p* = 0.06) but this was not reflected in incidence of RRT or days alive and free of RRT (Table 2). Other measures of organ failure and number of severe bleeding episodes were similar in the two groups. The 180-day mortality was lower in the dextran group than in the control group whereas mortality at 28- and 90 days did not differ between the groups (Table 2 and Fig. 2). For details concerning fluid administration and fluid balance please see Table 3. In summary the dextran group received less 5% and 20% albumin during the first 5 days in the ICU, the dextran group also received more crystalloids at day 1 in the ICU. The volume of packed red blood cells during the first five days in the ICU was higher than in the control group. Urinary output was lower and fluid balance more positive in the dextran group during the first days in the ICU compared to the control group. Number of patients in the dextran and control groups that experienced a bleeding episode did not differ between the groups (Table 2).

**Table 2:** Main outcome variables

	Propensity-matched groups		Relative risk (95% CI)	Absolute risk reduction (95% CI)	P^a^
Outcome	Control *n* = 245	Dextran *n* = 245			
AKIN max^b^ median (Q1-Q3^c^)	1 (0–3)	2 (0–3)			0.06
DAF^d^ of RRT, median (Q1-Q3)	28 (0–28)	28 (0–28)			0.52
DAF of vasopressors, median (Q1-Q3)	25 (0–27)	24 (0–26)			0.96
DAF of mechanical ventilation, median (Q1-Q3)	24 (0–28)	22 (0–27)			0.44
RRT^e^, no (%)	46 (18.8)	53 (21.6)	1.15 (0.81 to 1.64)	−2.9% (−9.9 to 4.2%)	0.42
Bleeding episodes^f^, no (%)	35 (14)	45 (18)	1.29 (0.86 to 1.93)	−4.1% (−10.6 to 2.5%)	0.21
28-day mortality, no (%)	86 (35.1)	78 (31.8)	0.91 (0.71 to 1.17)	3.3% (−5.1 to 11.7%)	0.41
90-day mortality, no (%)	109 (44.5)	96 (39.2)	0.88 (0.71 to 1.08)	5.3% (−3.4 to 14.0%)	0.21
180-day mortality, no (%)	123 (50.2)	102 (41.6)	0.83 (0.68 to 1.01)	8.6% (−0.2 to 17.4%)	0.046

^a^Wilcoxon rang sum or McNemar’s test

^b^Maximal Acute Kidney Injury Network classification score the first 10 days after admission

^c^Interquartile range

^d^Days Alive and Free

^e^Renal Replacement Therapy

^f^Defined by patients that received more than 3 units of packed red blood cells at any day the first 10 days after admission

**Table 3:** Fluid therapy

	Propensity score matched groups				
	Control, *n* = 245		Dextran, *n* = 245		P
	Median	Q1 to Q3	Median	Q1 to Q3	
Dextran-70, 60 mg/ml (ml)					
Day 1	0	0 to 0	1000	500 to 1000	<0.001
Day 2	0	0 to 0	0	0 to 500	<0.001
Day 3	0	0 to 0	0	0 to 0	<0.001
Mean per day^a^	0	0 to 0	399	200 to 654	<0.001
Albumin 50 mg/ml (ml)					
Day 1	0	0 to 500	0	0 to 500	0.17
Day 2	0	0 to 0	0	0 to 0	0.0.66
Day 3	0	0 to 0	0	0 to 0	0.80
Mean per day	99	0 to 253	50	0 to 219	0.03
Albumin 200 mg/ml (ml)					
Day 1	0	0 to 154	0	0 to 0	<0.001
Day 2	0	0 to 100	0	0 to 0	0.24
Day 3	0	0 to 100	0	0 to 81	0.71
Mean per day	43	0 to 121	17	0 to 80	<0.001
Crystalloids^b^ (ml)					
Day 1	2100	533 to 4000	3025	2000 to 4200	<0.001
Day 2	78	0 to 420	130	0 to 985	0.06
Day 3	0	0 to 200	27	0 to 300	0.09
Mean per day	1050	343 to 2104	1279	740 to 2162	0.13
Blood transfusion (ml)					
Day 1	0	0 to 328	250	0 to 600	<0.001
Day 2	0	0 to 0	0	0 to 295	0.008
Day 3	0	0 to 0	0	0 to 245	0.88
Mean per day	57	0 to 244	174	0 to 320	<0.001
Fluids in, total^c^ (ml)					
Day 1	4261	1942 to 6323	5700	3999 to 7475	<0.001
Day 2	2817	1852 to 3982	3699	2684 to 4645	<0.001
Day 3	2444	1253 to 3322	2924	2088 to 3964	0.006
Mean per day	3823	2413 to 4891	4544	3314 to 5600	<0.001
Urine output (ml)					
Day 1	1400	605 to 2545	1073	381 to 2076	0.009
Day 2	2243	1049 to 3622	1931	829 to 3211	0.04
Day 3	2640	1410 to 3850	2555	1025 to 3743	0.92
Mean per day	2010	376 to 3044	2078	327 to 3007	0.97
Total fluid balance^d^ (ml)					
Day 1	1989	0 to 4528	3516	1703 to 5732	<0.001
Day 2	0	−627 to 1550	995	0 to 2617	<0.001
Day 3	−1	−1361 to 323	−38	−896 to 841	0.11
Mean per day	581	−82 to 2015	1022	204 to 2264	0.03

The number of patients in the control group was: Day 1 = 245. Day 2 = 191. Day 3 = 138. Day 4 = 102. Day 5 = 79. The number of patients in the dextran group was: Day 1 = 245. Day 2 = 212. Day 3 = 175. Day 4 = 139. Day 5 = 124. The data were collected from the patient’s electronic charts. No missing values. The decreasing number of patients represents patients who died or were discharged from the ICU

^a^Mean per day represents mean fluid administration per day up to 5 days after admission. For patients with ICU-stay <5 days the mean per day was calculated for the length of stay

^b^Crystalloids represents the sum of NaCl 9 mg/ml and Ringer’s Acetate

^c^Fluids in, total represents the sum of all enteral and parenteral administered fluids including blood products

^d^Insensible perspiration not included

A pre-specified analysis was performed to investigate the interaction between dose of dextran-70 and outcome. In this analysis only patients receiving >900 ml dextran-70 the first 5 days after admission were included in the dextran group. The propensity score matching rendered 219 patients in the control group and 219 patients in the dextran group. The dose of dextran-70 in the dextran group the first day was 1000 ml (interquartile range 1000–1483 ml) and for the first 5 days in the treated patients was 413 ml/day (interquartile range 300–750 ml/day), and the cumulative dose for the ICU stay was 1500 ml (interquartile range 1000–2500 ml), which corresponds to 20 ml/kg (interquartile range 14–32 ml/kg). Matching produced well-matched groups and reduced standardized differences in baseline variables to ≤10% for all variables but age (Additional file 2). The differences between the groups in the secondary analyses were essentially unchanged compared to the primary analyses. For baseline data before and after propensity score matching and outcomes in the secondary analyses, see Additional files 2, 3, 4.

## Discussion

Mortality at 180 days was lower in the dextran group whereas mortality at earlier time points did not differ. The use of dextran-70 was associated with more transfusions and a more positive fluid balance compared to patients that only received crystalloids and albumin. No effect of dextran on number of severe bleeding episodes could be detected. A signal for worsening of AKI was detected in the dextran group but other measures of organ failure were similar in the dextran and control groups.

There is a paucity of high quality data regarding effects of dextran solutions on outcomes despite the fact that dextrans have been used clinically for more than 60 years. In the present study, propensity score matching was used in attempt to reduce bias and to estimate treatment effects of dextran-70. The finding that standardized differences were below 10% for all covariates included in the main analysis indicates that matching was successful in reducing imbalances between the treatment groups [30]. However, as discussed in more detail below, it must be stressed we cannot exclude that remaining imbalances in covariates, that were not accounted for in the propensity score model, may have influenced our results.

There are conflicting data with regard to effects of dextran-70 on renal function in patients suffering from septic shock. Thus it was recently reported that incidence of RRT in a cohort of patients with septic shock resuscitated with mainly Ringers acetate was lower than in historical controls resuscitated with a combination isotonic saline, albumin and dextran-70 (23% vs 48%) [23]. In contrast, a somewhat larger study using a similar design could not demonstrate a change in incidence of RRT by dextran-70 [22]. The present result of a similar incidence of RRT of about 20% in both the control and dextran groups does not provide support for adverse renal effects of dextran-70. It should be noted that the incidence of RRT in the present study is in the same range as that reported in several recent randomized controlled trials investigating effects of fluid therapy in sepsis and septic shock [12, 17, 18, 32]. In an attempt to increase sensitivity to detect changes in renal function that may be of importance for long-term mortality [33] we investigated maximum AKIN score during the first 10 days after admission. There was a signal for an increase in maximum AKIN score, which could indicate that dextran may be injurious to kidneys even if patient important outcomes such as events of RRT and days alive and free of organ failure were not affected. Alternatively this signal represents a chance finding.

Our finding that patients in the dextran group received more packed red blood cells during the 5 first days at the ICU, compared to patients in the control group is in line with the two previous studies investigating effects of dextran-70 in sepsis [22, 23]. However, in contrast to the above studies the present study could not demonstrate a difference in the number of episodes with severe bleedings between the dextran and control groups. Two mechanisms could be responsible for the increased transfusions of packed red cells in the dextran group. Firstly dextran-70 is suggested to induce a von Willebrand-like syndrome and [34] and to weaken fibrinogen polymerization [35, 36]. Weather these effects of dextran-70 increases clinically significant bleeding is unclear. Randomized trials were performed of dextran-70 versus crystalloids in the study of septic shock due to dengue in children [37] and a small study in shocked, adult trauma patients [38]. Neither of these trials reported increased bleeding with dextran-70. Nevertheless, increased bleeding incidence in the dextran group cannot be ruled out as the cause for the need of more blood transfusions in the present study. Secondly, dextran-70 is a more potent plasma expander than albumin and hemodilution will therefore occur to larger extent in dextran-70 resuscitated patients [7, 8, 37, 39]. Because hemoglobin level is controlled by the clinician, iatrogenic hemodilution is likely to contribute to the increased number of transfusions in the dextran group.

The finding that use of dextran-70 was associated with decreased use of albumin is in keeping with our hypothesis that dextran-70 can be used to reduce use of albumin. However, the use of dextran-70 was also associated with a more positive fluid balance during the first 5 days of admission. The positive balance could mainly be referred to the use of dextran-70 in excess of the relatively small reduction in volume of albumin and to a minor extent to the increase in transfusion of packed red cells (Table 3). This seems to be in in disagreement with the experimental and clinical data discussed above suggesting that dextran-70 is a better plasma expander than crystalloids, [7, 8, 37, 39] which would be expected to lessen the need for other fluids in dextran-70 group. This result may have several explanations. As mentioned above a more pronounced hemodilution by dextran-70 is likely to have led to transfusions contributing to a more positive fluid balance in the dextran group. Also, although the groups we carefully matched with regard physiological and laboratory parameters reflecting severity of illness, it is possible dextran-70 was more likely to be given to patients with more severe vascular leak and a higher need of fluid. Naturally it could be argued that a dextran-70 induced increase in vascular leak may have increased the volume requirements and contributed to the more positive fluid balance. However, dextrans are not known to influence on macromolecular permeability and have in fact been suggested to decease fluid permeability in experimental models [40, 41].

In attempt to evaluate if effects of dextran were dose-dependent we performed a second propensity score matching in which only patients receiving >900 ml dextran-70 were available for matching. This sensitivity analysis did not provide evidence for a dose dependent negative or positive effect of dextran-70 administration. Our finding that the effect of dextran on 90-day and 180-day mortality appeared to be more marked, aligns with the results of the main analysis. However, this finding may also be due to a statistical type 1 error since we did not correct for multiple testing and should be interpreted cautiously.

### Strengths

The strengths of the present study is that all physiological and laboratory variables and many pre-existing conditions were registered prospectively in electronic charts and collected as raw data directly from the electronic charts and not from a secondary electronic case report form or register. Taken together this makes the data robust and reliable. In addition, optimization of the propensity score matching was performed by an independent statistician without knowledge of outcomes prior to any comparisons between the groups.

### Limitations

Limitations include the single centre design and that the control group may not reflect practice in other ICUs, which makes the external validity of the study uncertain. Although baseline characteristics and comorbidities were carefully adjusted for it cannot be excluded that factors of importance for outcomes were not included in the propensity score model. Also, it cannot be excluded that patients in the control group may have received dextran-70 prior to arrival in the intensive care unit.

## Conclusions

No evidence to support a detrimental effect of dextran-70 on kidney function or need for organ support or mortality in patients with severe sepsis or septic shock could be detected. Treatment with dextran-70 was associated with increased transfusion of packed red cells and a more positive fluid balance in the first 5 days after admission but no support for an increased incidence of severe bleeding episodes was found. A prospective large trial with low risk of bias is needed to further evaluate effects of dextran-70 before it can be recommended as an alternative to albumin in the resuscitation of septic patients.

## Additional files

Additional file 1: Figure showing number of patients included in respective group each year. (DOCX 99 kb)

Additional file 2: Table showing patient demographics before and after propensity matching including only patients who received >900 ml dextran-70 during the first 5 days in the ICU in dextran group. (DOCX 106 kb)

Additional file 3: Table showing main outcome variables including only patients who received >900 ml dextran-70 during the first 5 days in the ICU in dextran group. (DOCX 20 kb)

Additional file 4: Figure showing Kaplan–Meier estimates of the probability of 180-day survival including only patients who received >900 ml dextran-70 the first 5 days in the ICU in dextran group. (DOCX 83 kb)

## Abbreviations

AKI: Acute kidney injury
AKIN: Acute Kidney Injury Network
COPD: Chronic obstructive pulmonary disease
DAF: Days alive and free of
HES: Hydroxylethyl starches
ICU: Intensive care unit
MV: Mechanical ventilation
RRT: Renal replacement therapy
SB: Spontaneous breathing
SIR: Swedish intensive care registry
