# Supplementary material for: Effect of dextran-70 on outcome in severe sepsis; a propensity-score matching study
Source: Scand J Trauma Resusc Emerg Med. 2017 Jul 6;25:65. doi: 10.1186/s13049-017-0413-x (PMC5501466; doi:10.1186/s13049-017-0413-x)
Supplement: Supplementary file 1 — Figure showing number of patients included in respective group each year. (DOCX 99 kb) [file 13049_2017_413_MOESM1_ESM.docx]

## Additional file 1
