# Supplementary material for: Effect of dextran-70 on outcome in severe sepsis; a propensity-score matching study
Source: Scand J Trauma Resusc Emerg Med. 2017 Jul 6;25:65. doi: 10.1186/s13049-017-0413-x (PMC5501466; doi:10.1186/s13049-017-0413-x)
Supplement: Supplementary file 2 — Table showing patient demographics before and after propensity matching including only patients who received >900 ml dextran-70 during the first 5 days in the ICU in dextran group. (DOCX 106 kb) [file 13049_2017_413_MOESM2_ESM.docx]

## Additional file 2. Patient demographics before and after propensity matching including only patients who received > 900 ml dextran-70 during the first 5 days in the ICU in dextran group.

|  | **Unmatched groups** | | | **Standardized difference** | **P-value** | **Propensity-matched groups** | | **Standardized difference** | **P-value** |
| --- | --- | --- | --- | --- | --- | --- | --- | --- | --- |
|  | **Control**  **N= 342** | **Dextran**  **N= 323** | |  |  | **Control**  **N= 219** | **Dextran**  **N= 219** |  |  |
| Pre-existing conditions | | | | | | | | | |
| Age, mean (SD^[[1]](#endnote-1)^) | 61.4 (17) | | 66.0 (17) | 0.29 | 0.0002 | 65.2 (14) | 63.2 (14) | 0.13 | 0.17 |
| Male gender, no (%) | 140 (41) | | 149 (46) | 0.10 | 0.18 | 91 (42) | 93 (42) | 0.02 | 0.85 |
| Blood malignancy^[[2]](#endnote-2)^, no (%) | 58 (17.0) | | 10 (3.1) | 0.17 | 0.03 | 10 (5) | 9 (4) | 0.02 | 0.82 |
| COPD^[[3]](#endnote-3)^, no (%) | 39 (11) | | 38 (12) | 0.011 | 0.88 | 32 (13) | 30 (14) | 0.001 | 0.89 |
| Chronic renal failure, no (%) | 14 (4.1) | | 8 (2.5) | 0.09 | 0.24 | 9 (4.1) | 7 (3.2) | 0.05 | 0.6 |
| Cirrhosis, no (%) | 15 (4.4) | | 7 (2.2) | 0.12 | 0.11 | 8 (3.7) | 7 (3.2) | 0.003 | 0.79 |
| Diabetes, no (%) | 38 (11) | | 8 (2.5) | 0.03 | 0.71 | 24 (11) | 26 (12) | 0.03 | 0.77 |
| Immunosuppression^[[4]](#endnote-4)^, no (%) | 54 (16) | | 32 (9.9) | 0.18 | 0.02 | 21 (9.6) | 23 (10) | 0.03 | 0.75 |
| Malignancy^[[5]](#endnote-5)^, no (%) | 46 (13) | | 44 (14) | 0.005 | 0.98 | 31 (14) | 29 (13) | 0.003 | 0.78 |
| Nosocomial infection^[[6]](#endnote-6)^, no (%) | 44 (13) | | 29 (9) | 0.12 | 0.11 | 23 (10) | 24 (11) | 0.01 | 0.88 |
| Surgery^[[7]](#endnote-7)^, no (%) | 71 (21) | | 69 (21) | 0.01 | 0.85 | 47 (21) | 47 (21) | 0.00 | 1.00 |
| GI^[[8]](#endnote-8)^ bleeding, no (%) | 3 (0.9) | | 2 (0.6) | 0.03 | 0.7 | 1 (0.5) | 2 (0.9) | 0.06 | 0.56 |
| DIC^[[9]](#endnote-9)^, no (%) | 39 (11) | | 21 (6.5) | 0.11 | 0.11 | 16 (7.3) | 17 (7.8) | 0.002 | 0.86 |
| I.C.^[[10]](#endnote-10)^ volume effect, no (%) | 5 (1.5) | | 0 (0) | 0.17 | 0.03 | 0 (0) | 0 (0) | 0.00 | 1.00 |
| Airway infection, no (%) | 94 (28) | | 79 (25) | 0.07 | 0.37 | 51 (23) | 61 (28) | 0.10 | 0.27 |
| Physiological and laboratory variables at admission^[[11]](#endnote-11)^, mean (SD ) | | | | | | | | | |
| Heart rate, mean (SD ) | 110 (24) | | 110 (25) | 0.03 | 0.68 | 110 (24) | 112 (24) | 0.07 | 0.46 |
| SBP^[[12]](#endnote-12)^, (mmHg) | 111 (30) | | 103 (30) | 0.27 | 0.0005 | 108 (31) | 107 (31) | 0.05 | 0.61 |
| Lactate (mmol/L) | 4.0 (3.8) | | 3.9 (3.8) | 0.03 | 0.67 | 3.6 (3.1) | 3.7 (3.1) | 0.02 | 0.84 |
| Norepinephrine (µg/min) | 3.7 (5.7) | | 5.2 (5.7) | 0.21 | 0.0006 | 4.1 (5.8) | 4.1 (5.8) | 0.003 | 0.98 |
| Temperature (°Celcius) | 37.3 (1.6) | | 37.3 (1.6) | 0.06 | 0.44 | 37.4 (1.4) | 37.3 (1.4) | 0.03 | 0.75 |
| Oxygenation points^[[13]](#endnote-13)^ | 2.0 (1.1) | | 1.9 (1.1) | 0.005 | 0.94 | 2.0 (1.1) | 2.1 (1.1) | 0.05 | 0.58 |
| Leucocytes (x 10^9^/L) | 16.6 (39.3) | | 13.5 (39) | 0.10 | 0.20 | 14.1 (14) | 13.9 (14) | 0.01 | 0.91 |
| Platelets (x 10^9^/L) | 151 (133) | | 184 (133) | 0.27 | 0.0006 | 178 (141) | 179 (141) | 0.007 | 0.94 |
| pH | 7.34 (0.14) | | 7.32 (0.14) | 0.14 | 0.07 | 7.34 (0.13) | 7.33 (0.13) | 0.07 | 0.44 |
| Bilirubin (µmol/L) | 31.0 (49.7) | | 20.9 (49.7) | 0.24 | 0.002 | 23.3 (23.4) | 23.9 (23.4) | 0.02 | 0.86 |
| Creatinine (µmol/L) | 173 (136) | | 181 (136) | 0.006 | 0.47 | 181 (129) | 190 (129) | 0.07 | 0.49 |

1. Standard deviation [↑](#endnote-ref-1)
2. Lymphoma, acute leukaemia or myeloma [↑](#endnote-ref-2)
3. Chronic obstructive pulmonary disease [↑](#endnote-ref-3)
4. Chronic steroid treatment correlative to ≥ 0.3 mg/kg prednisolone/day, radiation, or chemo therapy [↑](#endnote-ref-4)
5. Cancer spread beyond the regional lymph nodes [↑](#endnote-ref-5)
6. Infection that developed after ≥ 48 hours in hospital or secondary to surgical or medical procedure [↑](#endnote-ref-6)
7. Before admission to intensive care [↑](#endnote-ref-7)
8. Gastro-intestinal [↑](#endnote-ref-8)
9. Disseminated intravascular coagulopathy [↑](#endnote-ref-9)
10. Intra-cranial [↑](#endnote-ref-10)
11. First value within 90 min after admission except for “Norepinephrine” which is the mean dose until the first day's morning. [↑](#endnote-ref-11)
12. Systolic blood pressure [↑](#endnote-ref-12)
13. In accordance with SAPS 3. 1 point: PaO_2_ ≥ 8 kPa and spontaneous breathing (SB). 2 points PaO_2_ ≤ 8 kPa and SB. 3 points PaO_2_/FiO_2_ ≥ 13.3 and mechanical ventilation (MV). 4 points PaO_2_/FiO_2_ ≤ 13.3 and MV [↑](#endnote-ref-13)
