# Supplementary material for: Effect of dextran-70 on outcome in severe sepsis; a propensity-score matching study
Source: Scand J Trauma Resusc Emerg Med. 2017 Jul 6;25:65. doi: 10.1186/s13049-017-0413-x (PMC5501466; doi:10.1186/s13049-017-0413-x)
Supplement: Supplementary file 3 — Table showing main outcome variables including only patients who received >900 ml dextran-70 during the first 5 days in the ICU in dextran group. (DOCX 20 kb) [file 13049_2017_413_MOESM3_ESM.docx]

**Additional file 3. Main outcome variables including only patients who received > 900 ml dextran 70 the first 5 days in the ICU in dextran group**

|  | **Propensity-matched groups** | | **Relative risk (95% CI)** | **Absolute risk reduction (95% CI)** | **P^[[1]](#endnote-1)^** |
| --- | --- | --- | --- | --- | --- |
| **Outcome** | **Control**  **n= 219** | **Dextran**  **n= 219** |  |  |  |
| AKIN max^[[2]](#endnote-2)^, median (Q1-Q3^[[3]](#endnote-3)^) | 1 (0-3) | 2 (0-3) |  |  | 0.11 |
| DAF^[[4]](#endnote-4)^ of RRT, median (Q1-Q3) | 28 (0-28) | 28 (0-28) |  |  | 0.40 |
| DAF of vasopressors, median (Q1-Q3)^d^ | 25 (0-27) | 24 (0-26) |  |  | 0.97 |
| DAF of mechanical ventilation, median (Q1-Q3) | 24 (0-28) | 22 (0-27) |  |  | 0.51 |
| RRT^[[5]](#endnote-5)^, no (%) | 44 (20.1) | 51 (23.3) | 1.16 (0.81 to 1.66) | -3.2% (-10.9 to 4.5%) | 0.37 |
| Bleeding episodes^[[6]](#endnote-6)^, no (%) | 30 (13.6) | 38 (17.3) | 1.27 (0.81 to 1.97) | -3.7% (-10.4 to 3.1%) | 0.21 |
| 28-day mortality, no (%) | 74 (33.8) | 66 (30.1) | 0.89 (0.68 to 1.17) | 3.6% (-5.1 to 12.4%) | 0.40 |
| 90-day mortality, no (%) | 94 (42.9) | 78 (35.6) | 0.83 (0.66 to 1.05) | 7.3% (-1.8 to 16.4%) | 0.10 |
| 180-day mortality, no (%) | 105 (48.0) | 83 (37.9) | 0.79 (0.63 to 0.98) | 10.0% (0.8 to 19.2%) | 0.03 |

1. Wilcoxon rang sum or McNemar´s test [↑](#endnote-ref-1)
2. Maximal Acute Kidney Injury Network classification score the first 10 days after admission [↑](#endnote-ref-2)
3. Interquartile range [↑](#endnote-ref-3)
4. Days Alive and Free [↑](#endnote-ref-4)
5. Renal Replacement Therapy [↑](#endnote-ref-5)
6. Defined by patients that received more than 3 units of packed red blood cells at any day the first 10 days after admission [↑](#endnote-ref-6)
