# Supplementary material for: Effect of dextran-70 on outcome in severe sepsis; a propensity-score matching study
Source: Scand J Trauma Resusc Emerg Med. 2017 Jul 6;25:65. doi: 10.1186/s13049-017-0413-x (PMC5501466; doi:10.1186/s13049-017-0413-x)
Supplement: Supplementary file 4 — Figure showing Kaplan–Meier estimates of the probability of 180-day survival including only patients who received >900 ml dextran-70 the first 5 days in the ICU in dextran group. (DOCX 83 kb) [file 13049_2017_413_MOESM4_ESM.docx]

**Additional file 4**

**
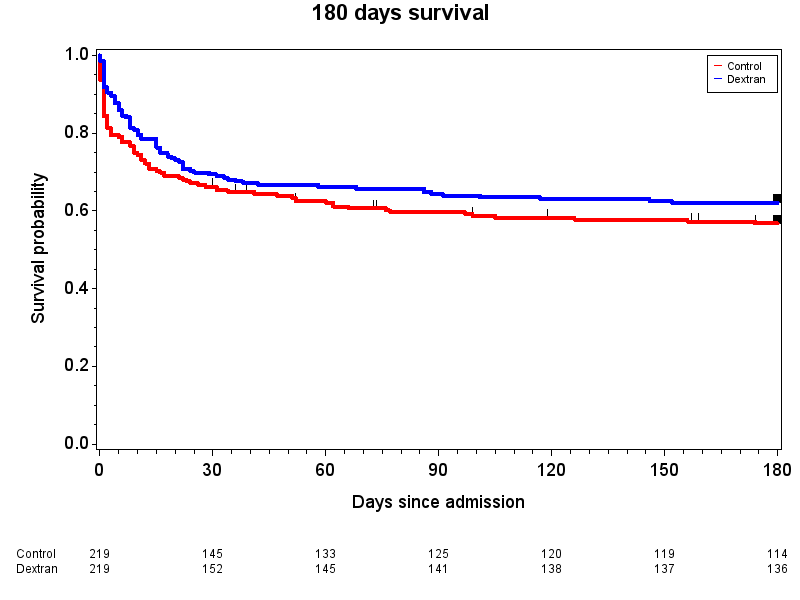
**

**Additional Fig. 2** Kaplan–Meier estimates of the probability of 180-day survival including only patients who received > 900 ml dextran 70 the first 5 days in the ICU in dextran group. P=0.19 for the comparison between the control group (red line) and the dextran group (blue line). Difference between groups was tested using the stratified log-rank test
